# Experimental and thermodynamic modeling decitabine anti cancer drug solubility in supercritical carbon dioxide

**DOI:** 10.1038/s41598-020-80399-7

**Published:** 2021-01-13

**Authors:** Mahboubeh Pishnamazi, Samyar Zabihi, Sahar Jamshidian, Fatemeh Borousan, Ali Zeinolabedini Hezave, Azam Marjani, Saeed Shirazian

**Affiliations:** 1 Institute of Research and Development, Duy Tan University, Da Nang, 550000 Viet Nam; 2 The Faculty of Pharmacy, Duy Tan University, Da Nang, 550000 Viet Nam; 3 Department of Process Engineering, Research and Development Department, Shazand-Arak Oil Refinery Company, Arak, Iran; 4 Environment, Development and Sustainability Department, Shadram Company, Arak, Iran; 5 Department of Chemistry, Yasouj University, Yasouj, 75914-353 Iran; 6 Incubation Centre of Science and Technology Park, Fanavari Atiyeh Pouyandegan Exir Company, Arak, 381314-3553 Iran; 7 Incubation Centre of Science and Technology Park, Fanavari Arena Exir Sabz Company, Arak, 381314-3553 Iran; 8 Department for Management of Science and Technology Development, Ton Duc Thang University, Ho Chi Minh City, Viet Nam; 9 Faculty of Applied Sciences, Ton Duc Thang University, Ho Chi Minh City, Viet Nam; 10 Laboratory of Computational Modeling of Drugs, South Ural State University, 76 Lenin prospekt, Chelyabinsk, Russia 454080

**Keywords:** Molecular medicine, Chemistry, Engineering, Materials science, Mathematics and computing

## Abstract

Design and development of efficient processes for continuous manufacturing of solid dosage oral formulations is of crucial importance for pharmaceutical industry in order to implement the Quality-by-Design paradigm. Supercritical solvent-based manufacturing can be utilized in pharmaceutical processing owing to its inherent operational advantages. However, in order to evaluate the possibility of supercritical processing for a particular medicine, solubility measurement needs to be carried out prior to process design. The current work reports a systematic solubility analysis on decitabine as an anti-cancer medicine. The solvent is supercritical carbon dioxide at different conditions (temperatures and pressures), while gravimetric technique is used to obtain the solubility data for decitabine. The results indicated that the solubility of decitabine varies between 2.84 × 10^–05^ and 1.07 × 10^–03^ mol fraction depending on the temperature and pressure. In the experiments, temperature and pressure varied between 308–338 K and 12–40 MPa, respectively. The solubility of decitabine was plotted against temperature and pressure, and it turned out that the solubility had direct relation with the pressure due to the effect of pressure on solvating power of solvent. The effect of temperature on solubility was shown to be dependent on the cross-over pressure. Below the cross-over pressure, there is a reverse relation between temperature and solubility, while a direct relation was observed above the cross-over pressure (16 MPa). Theoretical study was carried out to correlate the solubility data using several thermodynamic-based models. The fitting and model calibration indicated that the examined models were of linear nature and capable to predict the measured decitabine solubilities with the highest average absolute relative deviation percent (AARD %) of 8.9%.

## Introduction

It has been recognized that use of the current organic solvents in manufacturing processes causes a number of drawbacks such as adverse environmental impacts and high processing costs, especially in pharmaceutical industries that require high level of health standards. Therefore, the demands for green, non-organic, non-toxic, non-hazardous, cheap, and available solvents are rising worldwide to replace the current solvents^1^. An appropriate alternative for organic solvents in pharmaceutical manufacturing are supercritical solvents specially supercritical carbon dioxide which has attracted much attention recently in processing nanomedicines and crystallization of active pharmaceutical ingredients (APIs)^2–5^. Being non-toxic, moderate supercritical temperature and pressure, cheap solvent, and safety are the main advantages of carbon dioxide which have made this gas as the most common solvent for supercritical processing in pharmaceutical applications^6^.

Different techniques and processes have been developed and studied to assess the applicability of supercritical CO_2_ (SC-CO_2_) as green solvent in pharmaceutical industry such as rapid expansion^7,8^ and anti-solvent process^9,10^. These processes are mainly based on dissolution of a drug candidate in the supercritical solvent, and then removal of solvent from the solution to obtain sub-micron sized drug particles with enhanced dissolution properties. The size of drug particles can be controlled by optimization of process parameters as well as processing conditions, e.g. temperature and pressure. Prior to processing a drug candidate via supercritical technique, measuring the solubility of drug in the solvent at various temperatures and pressures is of great importance to assess whether the drug is appropriate for processing through this technology. Usually, gravimetric method is utilized to measure the solubility of APIs in supercritical solvents (e.g. CO_2_) by which the weight of drug before and after contacting with solvent is measured^11^.

There are several works in literature reporting the measurement of saturation solubility of various APIs in supercritical solvents at wide range of temperature and pressure. Zabihi et al.^12^ reported solubility of fenoprofen in supercritical CO_2_ at 308–338 K and 12–40 MPa. The solubility data was reported to be between 2.01 × 10^–5^ and 4.20 × 10^–3^ mol fraction depending on the pressure and temperature of the system. It was reported that there was a cross-over pressure point at 16 MPa, above which the temperature showed direct effect on the solubility. Pishnamazi et al.^13^ reported the measured and modelled solubilities of an anti-cancer drug (busulfan) in wide range of temperature and pressure in supercritical carbon dioxide. Experimental measurements of other drug candidates in supercritical CO_2_ have been reported in literature with focusing on evaluation of this technology for drug nanonization and bioavailability enhancement^11,14–18^.

In spite of experimental measurements of API solubility in supercritical CO_2_, some studies have been carried out to predict the drug solubility at different conditions in order to build a design space and predictive model. Different modeling approaches have been proposed for simulating the solubility of medicines using thermodynamic approaches^19–21^. Zabihi et al.^12^ reported that the solubility of fenoprofen in supercritical CO_2_ can be correlated with semi-empirical density-based correlations with the AARD% of 6%. Solubility of an API namely esomeprazole in CO_2_ at supercritical condition was correlated using equation of state approach at different conditions (12–27 MPa and 308.2–338.2 K)^22^. It was indicated that EoS models predicted the solubility data with AARD% of close to 8% for different circumstances. The solubility of lansoprazole as API in SC-CO_2_ was measured and simulated by Sodeifian et al.^23^ using EoS methods including Peng-Robinson and SAFT-VR. Indeed, either EoS or semi-empirical approaches can be employed in order to predict the solubility of APIs in SC-CO_2_ at different conditions, however semi-empirical approach is easier to implement and can be easily fitted using simple regression techniques^4,11,16^.

Considering the importance of measuring and predicting solubility of APIs in supercritical solvents, and given that there is no reports on the decitabine solubility, the current work is aimed to report a systematic approach for experimental measurements as well as modelling solubility of this API in wide range of temperature and pressure. Decitabine is known as an anti-cancer medicine, prescribed and taken during the chemotherapy stage and is generally classified as an antimetabolite and a demethylation agent to treat myelodysplastic syndromes^24^. Considering the side effects of this medicine, it is of great importance to prepare at sub-micron size or nano size to reduce the dosage and minimize its side effects. Therefore, solubility of decitabine is measured between 308–338 K, and 12–40 MPa in SC-CO_2_. Furthermore, the measured solubility data were correlated using five semi-empirical density-based correlations to build a predictive design space for the process development.

## Experiments

### Materials

Decitabine was supplied from Chem-Impex International, Inc., with the purity > 99% based on HPLC analytical test (see Table 1), and was further purified by SC-CO_2_ at the pressure of 500 bar and temperature of 338 K for a period of 3 h to separate any impurities^4,12^. The gaseous CO_2_ for the tests was supplied from a local company (purity > 99.8%).

**Table 1:** Properties of decitabine.

Molecular weight (g mol^−1^)	Chemical formula	Purity (HPLC)	Chemical structure
228.21	C_8_H_12_N_4_O_4_	99%	

### Laboratory setup

**Figure 1:**
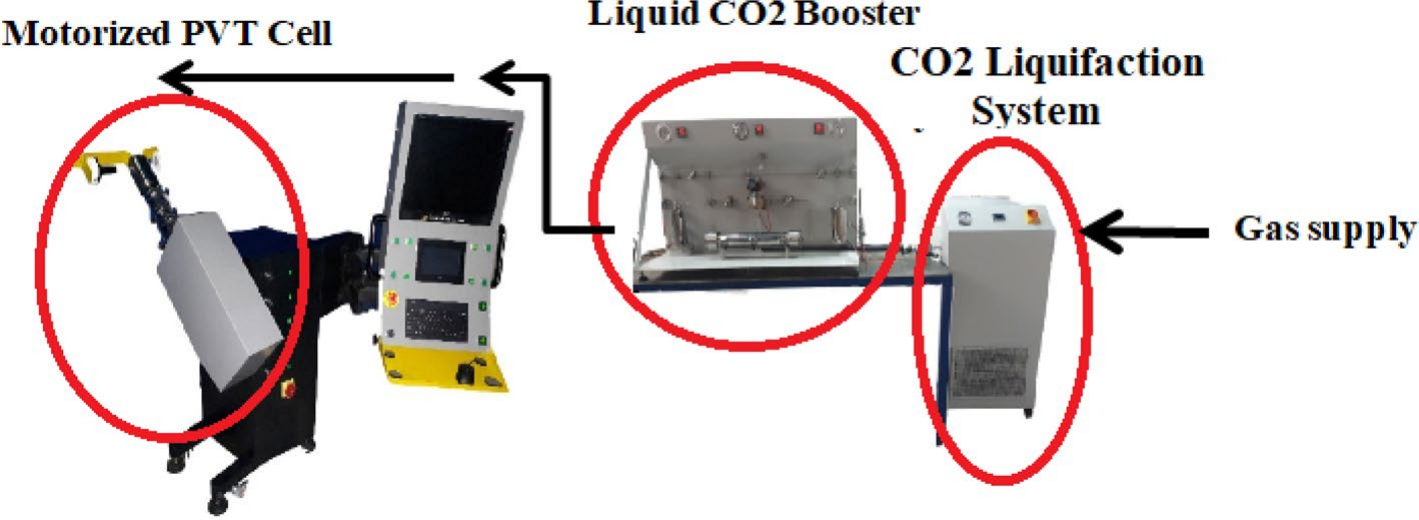
The supercritical based solubility apparatus used in this study. Reprinted with permission from^12^. Copyright [2020] *American Chemical Society*.

To determine the solubility of decitabine in the solvent (SC-CO_2_), an apparatus based on the static technique combined with gravimetric method was designed as illustrated in Fig. 1. The solubility of decitabine was obtained at pressures between 12–40 MPa, and temperatures of 308 to 338 K to evaluate the influence of temperature as well as pressure on the amount of decitabine solubility. As observed, the designed apparatus constitutes of two main parts, including condensation of solvent, and controlled solubility test chamber by varying Pressure, Volume, and Temperature (PVT cell). The volume of the PVT cell can be adjusted by using the integrated motorized pump, while the maximum volume that can be set is 0.4 L. The temperature and pressure of the cell is controlled by the PID controller connected to the chamber. In the condensation part of the setup, CO_2_ in converted to the condensate by decreasing the temperature down to 253 K, and then pressurizing via an air-driven reciprocating pump to the desired set-point pressure^4^. An inline filter and a surge tank are designed between the liquefaction unit and the PVT cell unit to prevent any damage to the cell and control system. It should be pointed out that one of the attractive advantages of the currently designed setup and method for solubility measurements is its simplicity, because this method only requires an accurate digital balance with a minimum accuracy of 0.1 mg and high-pressure and high-temperature vessel with known volume. So, it would be easier than the other available methods to measure the solubility of different APIs in SC-CO_2_ as a function of pressure and temperature^4,11,12,16,18^.

The weight of sample was measured before and after the test via a digital balance, and the solubility was obtained as:

1 $${\text{y}}_{{{2 }({\text{decitabine}})}} = {\text{ moles }}\;{\text{of }}\;{\text{decitabine}} / \, \left( {{\text{moles }}\;{\text{of}}\;{\text{ decitabine }} + {\text{ moles }}\;{\text{of}}\;{\text{ CO}}_{{2}} } \right)$$

The density values of CO_2_ were calculated at different pressures and temperatures similar to the method reported by Fat’hi et al.^25^. All measurements were performed in triplicate mode, and the average values are reported in this work.

### Modeling solubility data using semi-empirical density-based correlations

In order to simulate and predict the obtained solubility data for decitabine at different conditions, five models with semi-empirical basis were employed in this work. The models rely on density of solvent and can be easily fitted to the experimental data using regression techniques. These models have been used and validated in our previous studies for solubility of different types of API in supercritical CO_2_^4,12,13,16,18^. The models for predicting the solubility of decitabine in this work include: Chrastil^26^, Garlapati and Madras^20^, Mendez-Santiago and Teja (MST)^27^, Bartle et al.^28^, and Kumar and Johnston (K–J)^29^ models. These thermodynamic models have been well examined owing to their acceptable accuracy and simplicity which utilize only three fitting factors along with temperature, and density of CO_2_ which can be accurately estimated. The worth mentioning point in using these models for correlating decitabine solubility in SC-CO_2_ is that the models possess different fitting parameters because each one has been derived based on specific theory^30^.

Chrastil’s model with three fitting parameters can be expressed as:

2 $$\ln s{\text{ /kg}}\;{\text{m}}^{ - 3} = a + {\raise0.7ex\hbox{$b$} \!\mathord{\left/ {\vphantom {b {T/K}}}\right.\kern-\nulldelimiterspace} \!\lower0.7ex\hbox{${T/K}$}} + c \cdot \ln \rho /{\text{kg}}\;{\text{m}}^{ - 3}$$

where *a*, *b* and *c* are the fitting parameters which should be specified using curve fitting technique. *s* is the drug solubility, and *ρ* is the density of solvent at the experimental absolute temperature (*T*) and pressure^4^.

Chrastil’s model has been developed based on the theory of association in which it is assumed that each drug molecule has been surrounded by *c* molecules of solvent. Apart from the simplicity of this model in correlating the drug solubilities, one can estimate the enthalpies of vaporization and solvation as *ΔH*_*total*_/*R*, where *ΔH*_*total*_ is the summation of solvation and vaporization enthalpies of the solute, and *R* denotes the gas constant.

The correlation of Bartel et al.^28^ can be expressed as:

3 $$\ln \left( {\frac{{y_{{}} .p}}{{p^{ref} }}} \right) = a + {\raise0.7ex\hbox{$b$} \!\mathord{\left/ {\vphantom {b {T/K}}}\right.\kern-\nulldelimiterspace} \!\lower0.7ex\hbox{${T/K}$}} + c\cdot (\rho - \rho_{ref} )$$

where *y* is the drug solubility in the solvent (mole fraction). The superscript *ref* is the reference state which is considered 0.1 MPa for the pressure, and 700 kg m^−3^ for the density^31^.

The third model which is Mendez–Santiago–Teja (MST) can be written as:

4 $$T \cdot \ln \left( {\frac{y \cdot p}{{p^{ref} }}} \right) = a + b \cdot T/K + c \cdot \rho /{\text{kg}}\;{\text{m}}^{ - 3}$$

KJ^29^ and Garlapati and Madras^20^ models can be written as:

5 $$\ln y = a + {\raise0.7ex\hbox{$b$} \!\mathord{\left/ {\vphantom {b {T/K}}}\right.\kern-\nulldelimiterspace} \!\lower0.7ex\hbox{${T/K}$}} + c \cdot \rho \, /{\text{kmol}} {\text{m}}^{ - 3}$$

6 $$\ln y = a + b/{\text{T}}/{\text{K}} + c \cdot \ln \, (\rho /{\text{kg}} \;{\text{m}}^{ - 3} {\text{T}}/{\text{K}})$$

where, *a*, *b* and *c* are the fitting parameters.

## Results and discussions

**Figure 2:**
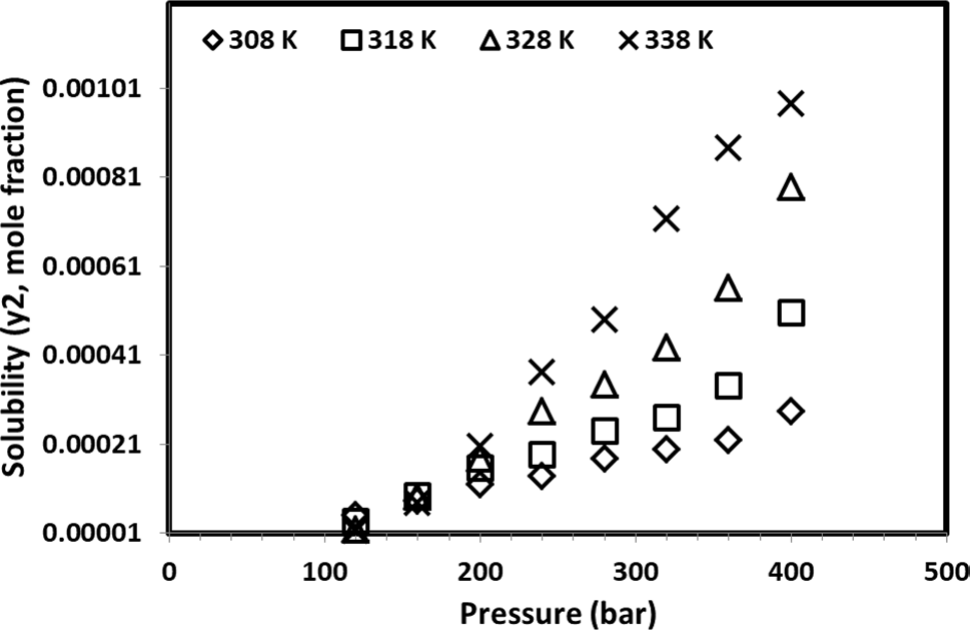
Measured solubility data for decitabine at different pressures and temperatures.

Decitabine solubility in the solvent in temperatures (308–338 K) and pressures (12–40 MPa) are indicated in Fig. 2 and Table 2. The measured solubility data are shown to be between 2.84 × 10^–5^ and 1.07 × 10^–3^ mol fraction with a calculated maximum relative standard deviation of about 8.90%. The measured solubility data revealed that it was possible to increase the solubility value of decitabine by rising the pressure regardless of the system’s temperature. In detail, for all examined isotherms, increasing the pressure leads to an enhancement in solubility of decitabine. Indeed, as the pressure increases the density of SC-CO_2_ is increased due to molecular compaction which directly enhances the solvating power of dense solvent, and more soluble drug would be obtained. But the effect of temperature on decitabine solubility is not straightforward, and the trend changes after the 160-bar pressure point.

**Table 2:** The solubility of decitabine as a function of temperature and pressure based on mole fraction (Standard uncertainties, u, are u (T) = 0.1 K and u (P) = 0.35 bar).

P/bar	T/K							
	308	u (y)	318	u (y)	328	u (y)	338	u (y)
120	5.04 × 10^–05^	4.51 × 10^–06^	4.51 × 10^–05^	3.86 × 10^–06^	3.69 × 10^–05^	2.66 × 10^–06^	2.84 × 10^–05^	2.35 × 10^–06^
160	8.23 × 10^–05^	6.55 × 10^–06^	9.37 × 10^–05^	6.90 × 10^–06^	9.11 × 10^–05^	7.79 × 10^–06^	7.79 × 10^–05^	6.26 × 10^–06^
200	1.18 × 10^–04^	6.14 × 10^–06^	1.55 × 10^–04^	1.21 × 10^–05^	1.77 × 10^–04^	1.27 × 10^–05^	2.05 × 10^–04^	1.43 × 10^–05^
240	1.37 × 10^–04^	1.72 × 10^–06^	1.87 × 10^–04^	9.45 × 10^–06^	2.82 × 10^–04^	1.63 × 10^–05^	3.71 × 10^–04^	1.71 × 10^–05^
280	1.76 × 10^–04^	1.20 × 10^–06^	2.40 × 10^–04^	8.49 × 10^–06^	3.42 × 10^–04^	2.00 × 10^–05^	4.90 × 10^–04^	2.27 × 10^–05^
320	1.97 × 10^–04^	5.81 × 10^–05^	2.69 × 10^–04^	2.85 × 10^–06^	4.27 × 10^–04^	7.82 × 10^–06^	7.15 × 10^–04^	1.06 × 10^–05^
360	2.18 × 10^–04^	8.39 × 10^–06^	3.40 × 10^–04^	2.71 × 10^–05^	5.60 × 10^–04^	1.82 × 10^–05^	8.74 × 10^–04^	2.06 × 10^–05^
400	2.83 × 10^–04^	7.17 × 10^–06^	5.06 × 10^–04^	4.13 × 10^–06^	7.88 × 10^–04^	1.06 × 10^–05^	1.07 × 10^–03^	1.20 × 10^–05^

It has been reported that there are two competing phenomena with regards to the influence of temperature on drug solubility. The density of solvent is reduced with increasing temperature because of higher energy of molecules which results in free movement of solvent molecules. The second phenomenon could be recognized as the change of solid sublimation pressure that can cause a positive influence on the solubility of drug in the solvent^4,12,18,30^. As such, the net effect of these two competing phenomena will determine whether the temperature has an increasing or decreasing effect on the drug solubility^4^. As seen in Fig. 2, one can define a threshold pressure called cross-over pressure above which the temperature enhancement has a positive influence on the solubility of decitabine due to the domination of sublimation pressure variation at higher temperatures. For the pressures below this point, increasing temperature leads to a reduction in the solubility of decitabine due to reduction of solvent density^16^. The existence of cross-over pressure has been observed in previous studies^32^, and can confirm the validity of the measurements in this work.

In the next stage of this research, the models introduced in Eqs. (2–6) were applied to correlate the obtained decitabine solubility data. The models’ unknown parameters (i.e. *a*, *b*, *c*) were estimated via multiple linear regression technique. The unknown parameters are reported in Table 3 with calculated AARD% (Average Absolute Relative Deviation) for each model to compare the correlative accuracy of individual models. It is seen that KJ (Kumar and Johnston) model with the lowest AARD % (9.04%) is the most accurate one, while the other models can correlate the solubility data with AARD % of between 13 and 15%. Additionally, using Bartle et al. and Chrastil models we calculated the value of *ΔH*_*total*_ about 61.54 kJ/mol and *ΔH*_*vap*_ about 80.73 kJ/mol. Also, *ΔH*_*sol*_ was calculated using Hess’s law which was equal to -19.19 kJ/mol.

**Table 3:** Fitting parameters and AARD % of the examined correlations.

Models (AARD %)^a^	Constants		
	*a*	*b*	*c*
Mendez–Santiago–Teja (13.3%)	− 12,862	27.145	3.825
Bartle et al. (15.3%)	25.758	− 9710.2	0.0119
Kumar and Johnston (9.04%)	7.8	− 7327.4	0.348
Chrastil (15.3%)	− 7402.5	23.225	6.84
Garlapati and Madras (15.3%)	−64.05	− 5513.7	5.83

^a^AARD % = 100 × ∑ ((y^calc^ − y^exp^)/y^exp^).

**Figure 3:**
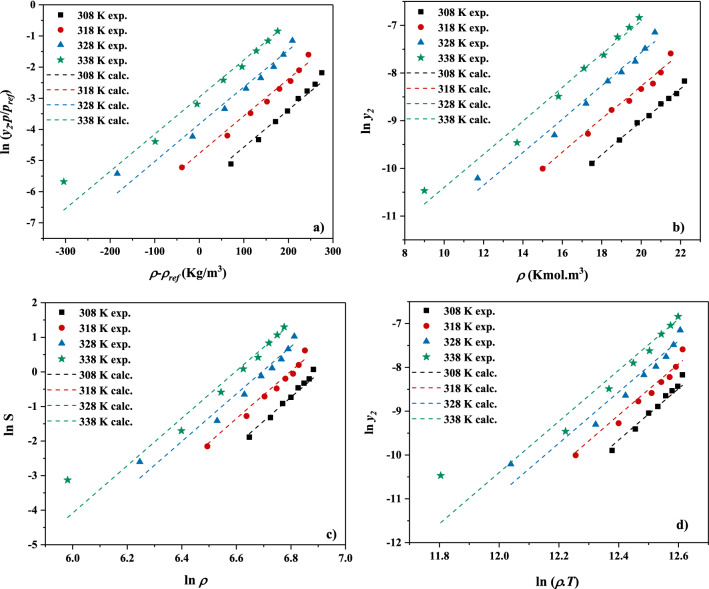
Correlated decitabine solubility using (**a**) Bartle et al. model, (**b**) KJ model, (**c**) Chrastil and (**d**) Garlapati and Madras model.

**Figure 4:**
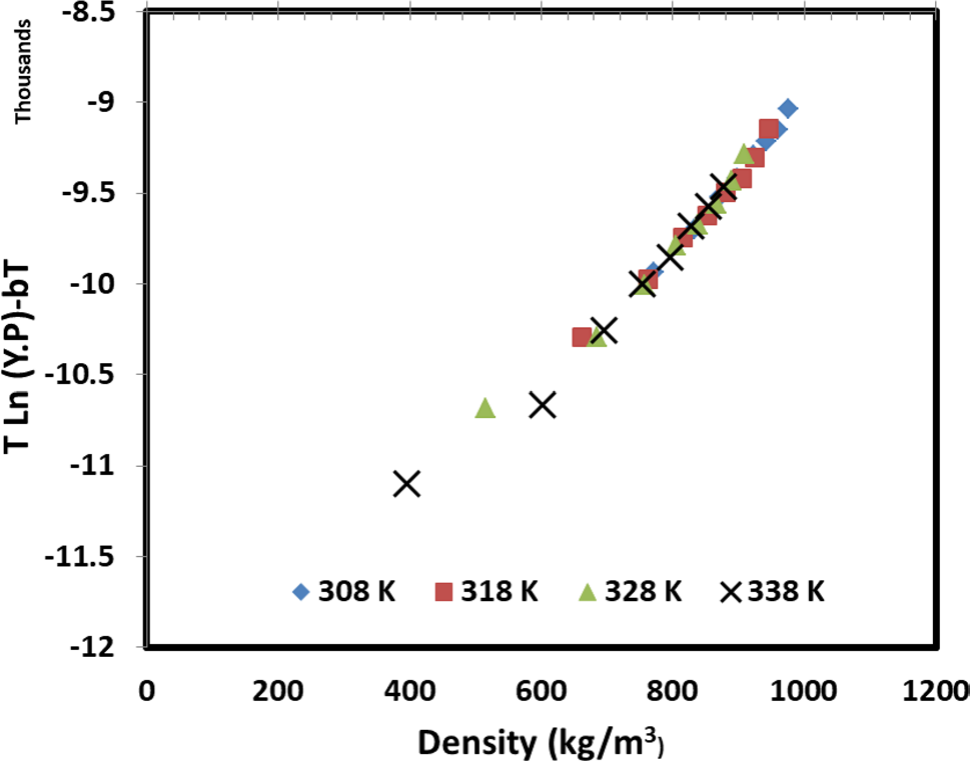
Extrapolative analysis based on MST equation.

Once the fitting parameters have been obtained, the models’ findings are plotted against the measured data, and the results are shown in Fig. 3 for four models. It is seen that the models offer almost the same behavior with linear trends, and great agreement has been obtained between the measured and correlated data for decitabine solubility in wide range of pressure and temperature. Furthermore, the possibility of using these models for extrapolation of the data was examined using self-consistency test for the MST model. Given that the examined 5 models showed almost similar behavior, MST model was selected for the self-consistency test as representative of the studied models in this work. The results are shown in Fig. 4. It is seen that the model shows extrapolative capability in predicting the solubility data out of the measured range, which is valuable advantage of the studied models for decitabine.

Furthermore, the self-consistency analysis using MST model (Fig. 4) turned out that the accuracy of the models is decreased at high levels of temperature. Indeed, as the temperature rises in the experiments the deviation from the linear behavior is increased which may be because of the significant effect of temperature on the sublimation pressure. The latter can directly affect the accuracy of the utilized correlations for decitabine solubility. Unfortunately, since solid–vapor equilibrium is a complex phenomenon especially for the systems dealing with supercritical state, it is challenging to find a generalized correlation and trend for wide range of temperature and pressure especially temperature since it has a dual effect on drug solubility as discussed before.

For more clarifications and understanding of the research outcomes in this work for broader applications, the performance of four models including Bartle et al., Mendez–Santiago–Teja, Kumar and Johnston, and Chrastil were analyzed using the solubility of different APIs, obtained from literature for various temperatures and pressures. The analyzed data revealed that there was no straightforward and generalized relationship between the accuracy of the correlations, API type, and operational conditions applied for the measurements. For instance, the calculated rosuvastatin, simvastatin, atorvastatin, lovastatin, and fluvastatin solubilities^33^ showed no specific correlation in terms of the molecular weight, model’s accuracy, and the considered temperature/pressure.

## Conclusions

Solubility of decitabine as an anti-cancer drug in supercritical CO_2_ using gravimetric method was reported in this work. The solubilities were obtained and reported between 308–338 K, and 12–40 MPa. The solubility data varied between 2.84 × 10^–5^ and 1.07 × 10^–3^ based on the mole fraction unit with indicated maximum relative standard deviation of 8.90%. It was observed that the relation between solubility and pressure was direct, while for temperature some complexities were observed. As such, the measurements revealed a cross-over pressure point of about 160 bar where for the pressures higher than this value, temperature and solubility showed a direct relation since sublimation pressure was dominant, while for the pressures lower than that value, the temperature had a negative effect on the decitabine solubility since density reduction is a more dominant phenomenon. Furthermore, the correlative and extrapolative capability of five semi-empirical density-based correlations were examined, and acceptable level of accuracy was obtained for KJ model with AARD% of 9.04%. Finally, the self-consistency analysis was performed for the MST model as a representative of these five examined correlations, and indicated that not only the evaluated correlations could correlate the solubility of decitabine in SC-CO_2_ as a function of temperature and pressure, but also it was possible to extrapolate the solubility data of decitabine in the pressures and temperatures out of the examined conditions in the current study.
